# Phytochemical Analysis Using UPLC-MS^n^ Combined with Network Pharmacology Approaches to Explore the Biomarkers for the Quality Control of the Anticancer Tannin Fraction of *Phyllanthus emblica* L. Habitat in Nepal

**DOI:** 10.1155/2021/6623791

**Published:** 2021-03-25

**Authors:** Lingfang Wu, Qiunan Zhang, Wenyi Liang, Yongben Ma, Liying Niu, Lanzhen Zhang

**Affiliations:** ^1^School of Chinese Materia Medica, Beijing University of Chinese Medicine, Beijing 100102, China; ^2^Hebei TCM Formula Granule Engineering and Technology Research Center, Hebei University of Chinese Medicine, Shijiazhuang 050091, China; ^3^Hebei TCM Quality Evaluation & Standardization Engineering Research Center, Shijiazhuang 050091, China

## Abstract

*Phyllanthus emblica* L. is widely used in traditional Tibetan medicine for its therapeutic effects on treating liver, kidney, and bladder problems. We have reported that the tannin fraction has a good anti-hepatocellular carcinoma effect, but its active ingredients are not clear. This study was to find the active ingredients of the tannin fraction using UPLC-MS^n^ and network pharmacology. First of all, the UPLC-MS^n^ method was employed to obtain high-resolution mass spectra of different components, and 110 compounds were obtained. Then a network pharmacology method was used to find biomarkers for quality control. Network pharmacology results showed that gallic acid, punicalagin A, punicalagin B, methyl gallate, geraniin, corilagin, chebulinic acid, chebulagic acid, and ellagic acid should be the biomarkers of the tannin fraction. Furthermore, 9 components were detected in the serum, which also proved that they could be biomarkers, because we generally believe that the ingredients which are absorbed into the blood are effective. In the end, a simple method for simultaneously determining the contents of the 9 compounds was constructed by HPLC-DAD. This research established a new method to find biomarkers of traditional Chinese medicine. This is of great significance to improving the quality standards of Tibetan medicine.

## 1. Introduction

Traditional Tibetan medicine has evolved from 2,300 years ago and still plays an important role in protecting human health. It is a vital part of traditional Chinese medicine. It can draw extensive attention for its mysterious nature and good effectiveness. *Phllanthus emblica* L. is widely used in traditional Tibetan medicine due to its numerous pharmacological applications in chronic diseases (for example, hypertension, hepatitis, blood stasis, and pharyngitis) [1–4]. It is an edible fruit indigenous to Southeast Asia and has been considered as a potent functional food. It is increasingly recognized that food and diet can maintain health and reduce the risk of chronic diseases.

*Phllanthus emblica* L. exhibits several biological effects, antioxidant [5, 6], anti-age [7], anticancer [8], anti-cardiovascular diseases [9], anti-diabetes [10], anti-inflammatory [6, 11], anti-microbial [12], anti-diarrheal [13], immune-modulating [14], hepato- and gastroprotective activities [15], analgesic activities [16], and so on. Also, hydrolysable tannins may be effective substances [17–20].

As part of our phytochemical investigation of medicinal plants for the discovery of new bioactive natural products, we have already reported the chemical constituents [17, 18] isolated from *Phyllanthus emblica* L., and the tannin fraction has good antitumor activity [19, 20]. We also established the stable preparation processes of the tannin fraction of *Phyllanthus emblica* L. However, most of the chemicals in the tannin fraction remain unknown, making it difficult to rationalize its bioactivity or evaluate the safety of this material as a therapeutic agent. Therefore, there is an urgent need to develop an analytical method capable of determining the chemical compositions in the tannin fraction.

The therapeutic effects of traditional Chinese medicines (TCM) are based on the complex interactions of complicated chemical constituents as a whole system. It is obviously unreasonable to use only a few ingredients for quality control. It is also necessary to associate ingredients with activity. Thus, choosing the right ingredients to reflect the quality of traditional Chinese medicine is the key issue. We researched the relevant literature on the quality control of the tannin fraction of *Phyllanthus emblica* L. Some scholars used HPLC to determine the content of a few compounds in *Phyllanthus emblica* L. [21, 22], but there was no correlation between ingredients and efficacy.

This research established a new method to find biomarkers for the quality control of traditional Chinese medicine. We firstly used the UPLC-MS^n^ method to obtain high-resolution mass spectra of the different components. A total of 110 compounds including 45 hydrolysable tannins, 22 mucic acids, 15 phenolic acids, 15 flavonoids, 11 organic acids, and 2 other compounds were tentatively identified by comparing their retention times and mass spectrometry data with those of the reference compounds and reviewing the literature. Then, a network pharmacology method was used to find biomarkers for quality control based on the 110 identified compounds and anti-hepatocellular carcinoma effect. Network pharmacology results showed that gallic acid, punicalagin A, punicalagin B, methyl gallate, geraniin, corilagin, chebulinic acid, chebulagic acid, and ellagic acid might be the biomarkers of the tannin fraction, and these 9 components were detected in the serum, which also proves that they could be biomarkers, because we generally believe that the ingredients those are absorbed into the blood are effective. In the end, a simple method for simultaneously determining the contents of the 9 compounds was constructed using HPLC-DAD. To the best of our knowledge, this is the first report using UPLC-MS^n^ and network pharmacology approaches to find the boimarkers for the quality control of the tannin fraction of *Phyllanthus emblica* L. The method developed in our study also provides a scientific foundation for the study of anticancer effective substances of the tannin fraction of *Phyllanthus emblica* L.

## 2. Materials and Methods

### 2.1. Samples and Reagents

Methanol (HPLC grade and MS grade) was purchased from Fisher Scientific (Waltham, MA, USA). Distilled water was purchased from Watson's Food & Beverage Co., Ltd. (Guangzhou, China). Acetic acid (MS grade) was purchased from Fisher Scientific (Waltham, MA, USA). Reference standards of gallic acid (98%, CAS No: 149-91-7), punicalagin A (63%, CAS No: 65995-63-3), punicalagin B (37%, CAS No: 65995-63-3), methyl gallate (98%, CAS No: 99-24-1), geraniin (98%, CAS No: 2360976-49-0), corilagin (98%, CAS No: 23094-69-1), chebulinic acid (98%, CAS No: 18942-26-2), chebulagic acid (98%, CAS No: 23049-71-5), and ellagic acid (98%, CAS No: 476-66-4) were purchased from Chengdu-PUSH Bio-Technology Co., Ltd. (Chengdu, Sichuan, China).

### 2.2. Plant Materials and Sample Preparation

*Phyllanthus emblica* L. was purchased from Tibet and authenticated by Professor Chun-Sheng Liu (School of Chinese Materia Medica, Beijing University of Chinese Medicine, Beijing, China). Voucher specimens (PE001) of the plant were deposited at the authors' laboratory. The crude drug was extracted with ethanol and separated by HPD-400 macroporous resin column chromatography. The sample was dried and powdered, before being sieved through a 40 mesh sieve. A sample of the powder (approximately 25 mg) was suspended in 50 mL of methanol, and the resulting mixture was filtered through a 0.22 *μ*m PTFE syringe filter. The filtrate was collected and subjected to centrifugation (13,000 rpm, 10 min). The supernatant was then transferred to an autosampler vial for analysis by UPLC-MS/MS and HPLC-DAD.

### 2.3. Apparatus and Parameters

The LTQ-Orbitrap XL UPLC-MS/MS instrument (Thermo Fisher, USA) was equipped with an ESI source used in negative ionization mode. The interface and MS parameters were as follows: nebulizer pressure, 100 kPa; dry gas, N_2_ (1.5 L/min); drying gas temperature, 200°C; spray capillary voltage, 4000 V; scan range, *m*/*z* 100–1500. Mobile phase: A (methanol); B (H_2_O : CH_3_COOH, 100 : 0.2, *v*/*v*). Column: ACQUITY UPLC BEH C18 1.7 *μ*m (2.1 × 100 mm, Column; Part No: 1860023452; Serial No: 0246325825758), maintained at 30°C with flow rate of 0.3 mL·min^−1^. The injection volume was 5 *μ*L. Gradient elution procedure: 0 min (5% A) ⟶ 5 min (15% A) ⟶ 8 min (25% A) ⟶ 10 min (30% A) ⟶ 18 min (60% A) ⟶ 26 min (90% A) ⟶ 34 min (90% A).

A Waters Alliance HPLC 2695 series instrument (Waters, Manchester, UK) was used to perform the high-performance liquid chromatography (HPLC) analysis. Mobile phase: A (methanol); B (H_2_O : CH_3_COOH, 100 : 0.2, *v*/*v*). Column: Diamansil^TM^ C18 (250 × 4.6 mm, 5 *μ*m), maintained at 30°C with flow rate of 1.0 mL·min^−1^. The detection wavelength was set at 270 nm for acquiring chromatograms. The injection volume was 20 *μ*L. Gradient elution procedure: 0 min (5% A) ⟶ 10 min (15% A) ⟶ 15 min (25% A) ⟶ 30 min (30% A) ⟶ 50 min (60% A) ⟶ 55 min (90% A) ⟶ 62 min (90% A).

### 2.4. Optimization of Analytical Conditions

To obtain better chromatographic separation and mass spectrometric detection, we evaluated three different mobile phase systems, including aqueous methanol, aqueous acetonitrile, and aqueous acetonitrile-formic acid solutions. The aqueous methanol solution resulted in the best separation of the major components of the tannin fraction of *Phyllanthus emblica* L. Furthermore, the addition of 0.2% acetic acid to this mobile phase resulted in a considerable improvement in the symmetry properties of the most chromatographic peaks. We also varied the flow rate (0.8, 1.0, and 1.2 mL/min) for HPLC analysis and (0.25, 0.3, and 0.35 mL/min) UPLC analysis, column temperature (25, 30, and 35°C) for HPLC and UPLC analysis, and injection volume (3, 5, and 10 *μ*L) for UPLC analysis during method development. The results of these optimization experiments established the following conditions for the chromatographic separation of the different components of the tannin fraction of *Phyllanthus emblica* L.

### 2.5. Structure Analysis Procedure

In the negative scan mode, based on the high-accuracy precursor ions and product ions obtained from UPLC-MS/MS, the elemental compositions were calculated when the maximum tolerance of mass error for the precursor ions and product ions was set at 1.5 ppm, which can satisfy the requirements for positive identification. Based on the elemental compositions of the precursors, the most rational molecular formula was sought in different chemical databases such as the Spectral Database for Organic Compounds SDBS, m/z cloud, and ChemSpider. Meanwhile, by searching literature sources, such as PubMed of the U.S. National Library of Medicine and the National Institutes of Health, Scifinder Scholar of the American Chemical Society, Science Direct of Elsevier, and Chinese National Knowledge Infrastructure (CNKI) of Tsinghua University, all components reported in the literature on *Phyllanthus emblica* L. and plants from the same family were summarized in a Microsoft Office Excel table to establish an in-house library [5, 7–13, 23] for searching the most rational molecular formula. When several matching compounds with the same formula were found, the fragmentation patterns and pathways of the compounds were analyzed and then validated by Mass Frontier 7.0 (Thermo Scientific) for positive identification.

### 2.6. Biomarkers Selected by Network Pharmacology and Ingredients Absorbed into the Blood

We followed the methods of Luo et al. 2020 [24]. Firstly, a network pharmacology method was used to find biomarkers for quality control based on the compounds identified by UPLC-MS^n^ and anti-hepatocellular carcinoma effect. Then to confirm that these compounds were proper quality control markers, animal experiments were conducted, with rats as test animals. We check whether these active ingredients are absorbed into the blood, because we generally believe that the ingredients those are absorbed into the blood are effective. The use of animals in the present study was permitted by the Ethics Committee of Beijing University of Chinese Medicine, and all animal studies were carried out according to the Guide for Care and Use of Laboratory Animals.

## 3. Results

### 3.1. Identification of the Compounds Present

**Figure 1:**
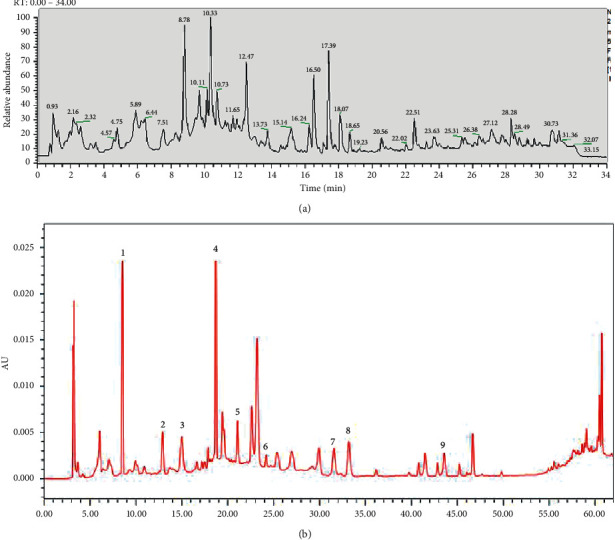
UPLC-MS^n^ chromatogram of the tannin fraction of *Phyllanthus emblica* (L) in the negative mode (a); HPLC chromatogram of the tannin fraction of *Phyllanthus emblica* (L) (b) gallic acid (1), punicalagin A (2), punicalagin B (3), methyl gallate (4), geraniin (5), corilagin (6), chebulinic acid (7), chebulagic acid (8), and ellagic acid (9).

**Figure 2:**
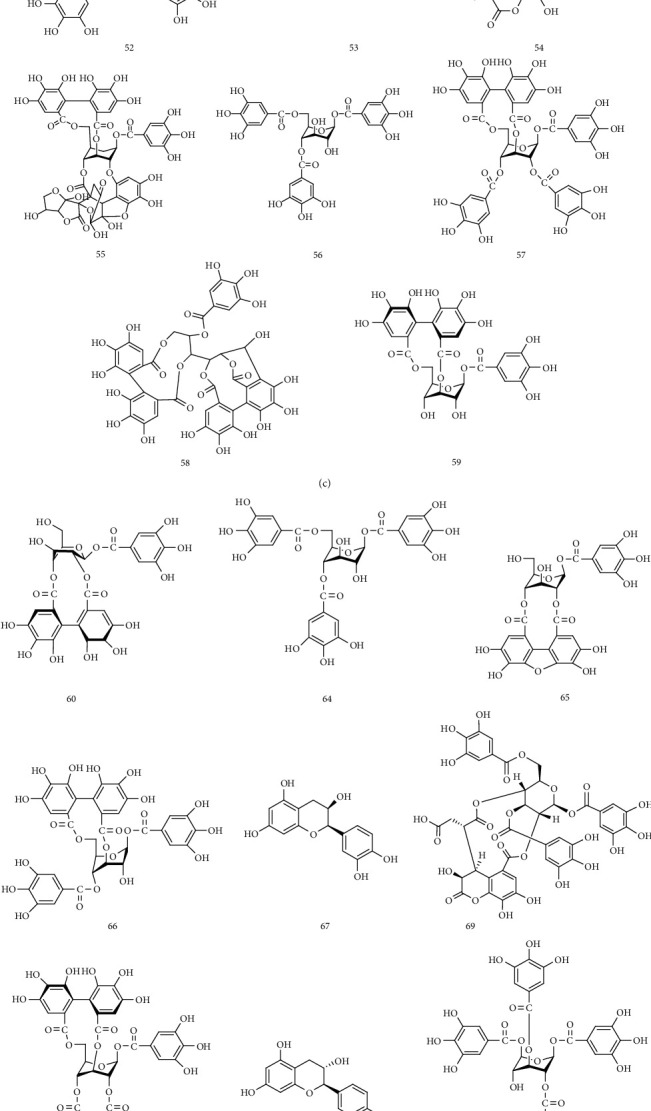
Structures of chemical constituents in the tannin fraction of *Phyllanthus emblica* L.

UPLC-MS/MS method was employed to identify the components in the tannin fraction of *Phyllanthus emblica* L. The total ion chromatogram profile of the tannin fraction of *Phyllanthus emblica* L. was presented in negative mode, as shown in Figure 1(a). Molecular weights and fragmentation information (Table 1) were obtained. The possible structures of all peaks were deduced as shown in Figure 2. Under the optimized MS conditions, the negative mode was used to identify the peaks. 110 compounds including 45 hydrolysable tannins, 22 mucic acids, 15 phenolic acids, 15 flavonoids, 11 organic acids, and 2 other compounds have been tentatively identified by comparing their retention times and mass spectrometry data with that of reference compounds and reviewing the literature. Data for all of these compounds are summarized in Table 1.

#### 3.1.1. Identification of Hydrolysable Tannins

45 hydrolysable tannins have been identified in the tannin fraction of *Phyllanthus emblica* L., accounting for more than 41% (45/110). As shown in Table 2, in the negative mode ESI-MS^1^ spectra, the [M−H]^−^ ion was observed for all compounds. In the negative mode ESI-MS^2^ spectra, the [M−galloyl−H]^−^ ion was observed for 36 compounds, such as compounds 10, 17, 19, 21, 25, 26, 27, 31, 38, 42, 47, 49, 50, 54, 55, 56, 57, 58, 59, 60, 61, 62, 63, 64, 65, 66, 71, 74, 75, 76, 77, 78, 79, 86, 88, and 102. The [M−2 galloyl−H]^−^ ion was observed for 18 compounds, such as compounds 17, 19, 23, 25, 26, 38, 48, 56, 57, 62, 64, 66, 71, 74, 75, 76, 78, and 88. The [2M−H]^−^ ion was observed for 9 compounds, such as compounds 27, 31, 36, 46, 47, 52, 69, 70, and 76. The [M−galloyl−HHDP−H] ^−^ ion was observed for 6 compounds, such as compounds 47, 49, 55, 60, 61, and 86. The [M−HHDP−H]^−^ ion was observed for 4 compounds, such as compounds 35, 36, 47, and 101. The [M−Ela−H]^−^ ion was observed for 2 compounds, such as compounds 39 and 41. The [M−3 galloyl−H]^−^ ion was observed for 1 compound, compound 88. The [M−THBDF−H]^−^ ion was observed for 1 compound, compound 65. From this result, it can be seen that [M−H]^−^ and [M−galloyl−H]^−^ are the most fragmented ions, followed by [M−2 galloyl−H]^−^ ions and [2M−H]^−^ ions.

#### 3.1.2. Identification of Mucic Acids

22 mucic acids have been identified in the tannin fraction of *Phyllanthus emblica* L., accounting for more than 20% (22/110). In the negative mode ESI-MS1 spectra, the [M−H]^−^ ion was observed for all the compounds. In the negative mode ESI-MS2 spectra, the [M−galloyl−H]^−^ ion was observed for 8 compounds, such as compounds 7, 12, 14, 16, 24, 28, 34, and 37. The [2M−H]^−^ ion was observed for 5 compounds, such as compounds 4, 24, 28, 34, and 37. The [M−2CH_2_−H]^−^ ion was observed for 3 compounds, such as compounds 18, 20, and 22. The [M−H_2_O−H]^−^ ion was observed for 3 compounds, such as compounds 3, 5, and 7. The [M−CO_2_−H]^−^ ion was observed for 2 compounds, such as compounds 3 and 4. The [M−2CO_2_−H]^−^ ion was observed for 1 compound, compound 4. From this result, it can be seen that [M−H]^−^ and [M−galloyl−H]^−^ are the most fragmented ions, followed by [2M−H]^−^ ions.

#### 3.1.3. Identification of Phenolic Acids and Phenolic Acid Glycosides

15 phenolic acids and their glycosides have been identified in the tannin fraction of *Phyllanthus* emblica L., accounting for about 14% (15/110). In the negative mode ESI-MS1 spectra, the [M−H]^−^ ion was observed for all compounds. In the ESI-MS2spectra, the [M−galloyl−H]^−^ ion was observed for 4 compounds, such as compounds 6, 9, 13, and 43. The [2M−H]^−^ ion was observed for 4 compounds, such as compounds 6, 9, 13, and 83. The [M−A−H]^−^ ion was observed for 4 compounds, such as compounds 33, 68, 72, and 80. (A represents various sugar groups). The [M−CO_2_−H]^−^ ion was observed for 2 compounds, compounds 15 and 33. The [M−2CO_2_−H]^−^ ion was observed for 1 compound, compound 29. The [M−CH_3_−H]^−^ ion was observed for 1 compound, compound 53. The [M−H_2_O−H]^−^, [M−CO−H]^−^ and [M−CO−CO_2_−H]^−^ ion was observed for 1 compound, compound 85. From this result, it can be seen that [M−H]^−^ and [M−galloyl−H]^−^ are the most fragmented ions, followed by [M−A−H]^−^ ions and [2M−H]^−^ ions.

#### 3.1.4. Identification of Flavonoids

15 flavonoids have been identified in the tannin fraction of *Phyllanthus* emblica L., accounting for about 14% (15/110). In the negative mode ESI-MS1 spectra, the [M−H]^−^ ion was observed for all compounds. In the negative mode ESI-MS2 spectra, the [M−A−H]^−^ ion was observed for 6 compounds, such as compounds 81, 84, 90, 91, 98, and 99 (A represents various sugar groups). The [M−galloyl−H]^−^ ion was observed for 2 compounds, such as compounds 77 and 102. The [M−CO_2_−H]^−^ ion was observed for 1 compound, compound 89. The [M−CH_2_−H]^−^ ion was observed for 1 compound, compound 67. The [M−CH_3_−H]^−^ ion was observed for 1 compound, compound 95. It can be seen that [M−H]^−^ and [M−A−H]^−^ are the most fragmented ions, followed by [2M−H]^−^ ions.

#### 3.1.5. Identification of Fatty Acid

11 fatty acids have been identified in the tannin fraction of *Phyllanthus emblica* L., accounting for about 10% (11/110). In the negative mode ESI-MS^1^ spectra, the [M−H]^−^ ion was observed for all compounds. In the ESI-MS^2^ spectra, the [M−H_2_O−H]^−^ ion was observed for 6 compounds, such as compounds 93, 97, 103, 106, 107, and 108. The [M−CO_2_−H]^−^ ion was observed for 6 compounds, such as compounds 1, 11, 100, 105, 109, and 110. The [M−CO_2_−H_2_O−H]^−^ ion was observed for 4 compounds, such as compounds 11, 103, 106, and 107. The [M−CH_3_−2CO_2_−H]^−^ ion and [M−CH_3_−H]^−^ ion were observed for compound 92. From this result, it can be seen that [M−H]^−^ and [M−H_2_O−H]^−^ are the most fragmented ions, followed by [M−CO_2_−H]^−^ ions.

### 3.2. Biomarkers Selected by Network Pharmacology and Ingredients Absorbed into the Blood

**Figure 3:**
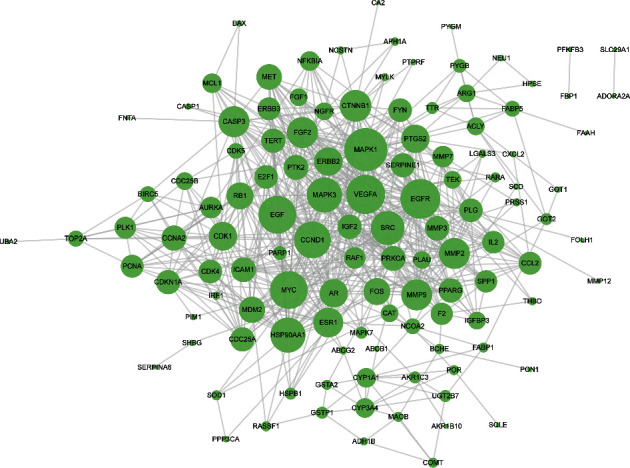
PPI network of “compound-target” in the tannin fraction of *Phyllanthus emblica* L. The bigger the graph, the bigger the degree. PPI: protein-protein interaction.

**Figure 4:**
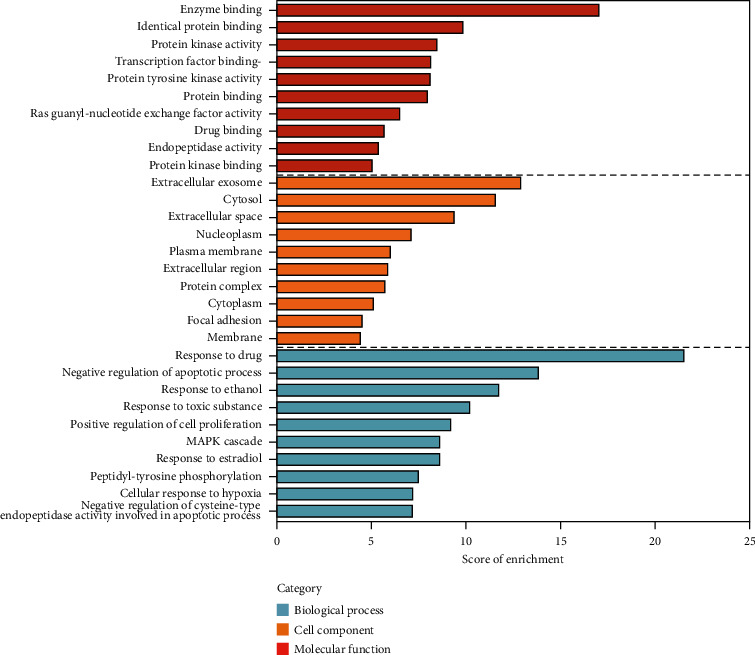
GO analysis of potential target genes of the tannin fraction of *Phyllanthus emblica* L. GO: gene ontology.

**Figure 5:**
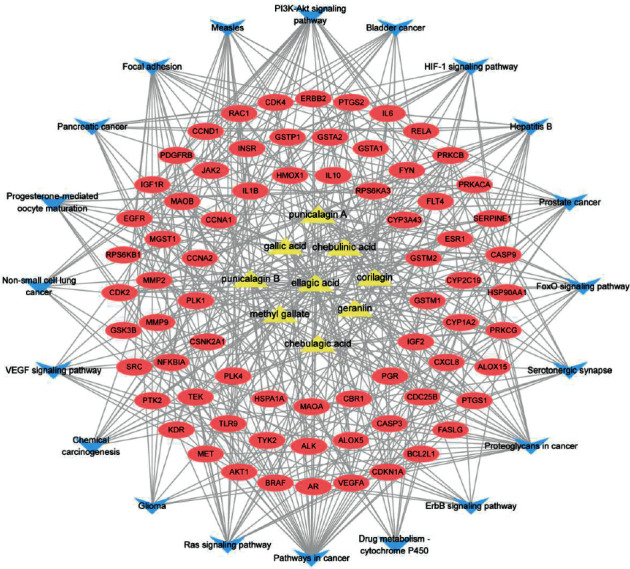
“Component-target-function” network. Note: yellow triangle: compounds; pink ellipse: target; blue triangle: biological process.

228 potential targets related to the 110 compounds were obtained by using Swiss Target Prediction and TCMSP databases. And 7392 potential targets related to hepatocellular carcinoma were obtained according to OncoDB.HCC and Liverome databases. Through protein-protein interaction analysis, 120 targets with higher correlation were obtained, as shown in Figure 3. The DAVID database was used to conduct GO enrichment analysis on 120 targets with *p*-value less than 0.01, as shown in Figure 4. Finally, the Cytoscape 3.7.1 software was used to visualize the “component-target-function” network, as shown in Figure 5. 9 compounds, 72 proteins, and 20 pathways were obtained. Of the 20 pathways, PI3K-Akt signaling pathway, HIF-1 signaling pathway, Ras signaling pathway, ErbB signaling pathway, FoxO signaling pathway, and VEGF signaling pathway are related to anticancer effect [25–31], these pathways may be related to the anti-cancer effect of the tannin fraction of *Phyllanthus emblica* L. And the 9 compounds including gallic acid, punicalagin A, punicalagin B, methyl gallate, geraniin, corilagin, chebulinic acid, chebulagic acid, and ellagic acid were all detected in the rat serum by using UPLC-MS/MS, which further verified that these compounds were proper biomarkers. Detailed information about the analysis of chemical components in rat serum can be found in the supplementary materials.

### 3.3. Validation of the HPLC Method

The method was validated in terms of linearity, precision, stability, repeatability, and recovery test.

The concentrations of gallic acid, punicalagin A, punicalagin B, methyl gallate, geraniin, corilagin, chebulinic acid, chebulagic acid, and ellagic acid in the stock solution were 0.09360, 0.1152, 0.2107, 0.0932, 0.3200, 0.5500, 0.1096, 0.08400, and 0.1800 *μ*g *μ*L−1, respectively. The stock solution was then diluted to appropriate concentration range for the establishment of calibration curves. The calibration curves were constructed by plotting the peak area (*Y*) versus the concentration (*X*, *μ*g) of each standard solution. Detailed information regarding the calibration curves and linear ranges is listed in Table 2. All calibration curves showed good linear regression within the test ranges.

The precision was determined by replicate injection with the same sample solution six consecutive times. The RSDs of peak area of gallic acid, punicalagin A, punicalagin B, methyl gallate, geraniin, corilagin, chebulinic acid, chebulagic acid, and ellagic acid were all below 3.05%, which showed high precision.

Stability testing was performed with one sample over 24 h. The RSDs of peak area of the 9 constituents were all below 2.71%, which indicated that the samples remained stable during the testing period and the conditions for the analysis were satisfactory.

The repeatability was evaluated by the analysis of six prepared samples. The RSDs of for the contents of 9 constituents were all below 3.61%, which showed high repeatability.

The recovery was determined by the standard addition method. Certain amounts of the 9 constituents were spiked into the known sample and then processed and quantified in accordance with the established procedures as shown in Sections 2.2 and 2.3. The average recoveries were between 98.11% and 103.16%, with RSD values of less than 3.01% for the 9 compounds. Therefore, the developed method was precise and sensitive enough for simultaneously quantitative analysis of 0 compounds in the tannin fraction of *Phyllanthus emblica* L.

### 3.4. Quality Evaluation of the 9 Compounds

The developed quantitative analysis method was subsequently applied to 6 batches of the tannin fraction of *Phyllanthus emblica* L. sample habitat in Nepal. The results demonstrated a successful application of this HPLC-DAD assay for the quantification of 9 major constituents in different samples. The 9 compounds have been eluted within 62 min, giving good separation and acceptable tailings factors. Representative HPLC-DAD chromatograms of standard solutions and sample solutions for quantitative analysis are shown in Figure 1. The contents, summarized in Table 3, were calculated with the external standard methods.

In this experiment, UPLC-MS^n^ was employed to analyze the tannin fraction of *Phyllanthus emblica* L. The total ion chromatograms under both positive and negative modes were investigated at first, but the response intensity in the negative mode was significantly increased, and the number of detected chromatographic peaks increased significantly. Therefore, the negative mode was selected for the detection of *Phyllanthus emblica* L. We tentatively identified a total of 110 compounds including 45 hydrolysable tannins, 22 mucic acids, 15 phenolic acids, 15 flavonoids, 11 organic acids, and 2 other compounds. It can be seen from this result that most of the compounds in the tannin fraction are hydrolysable tannins, and the number of compounds accounted for more than 41% (45/110). The total tannins in the tannin fraction were determined before, and the content reached more than 60%. It is consistent with the results detected by UPLC-MS^n^. There are also some mucic acids, phenolic acids, and flavonoids. Next, we will pay attention to these chemical components; the total content of flavonoids, mucic acids, phenolic acids, and organic acids accounts for about 40%; these ingredients may work synergistically with the hydrolysable tannins.

From the results of content determination by HPLC-DAD, the contents of gallic acid (content: 3.42%) and ellagic acid (content: 3.21%) are significantly higher than some hydrolysable tannins (punicalagin A: 0.26%, punicalagin B: 0.42%, chebulinic acid: 0.44%). Analyzing the reasons, we speculate that gallic acid and ellagic acid may be produced by the decomposition of other hydrolysable tannins. As we all know, gallic acid and ellagic acid are the basic structural units of hydrolysable tannins. Hydrolysable tannins are unstable; they are easily decomposed under acids, alkali, enzyme, and high temperatures and used to produce gallic acid, ellagic acid, and polyols. In the process of preparing tannin fraction, the extraction temperature is 60°C and some hydrolysable tannins may decomposed, and these need to be further confirmed.

## 4. Conclusions

This research established a new method to find biomarkers for quality control of the tannin fraction of *Phyllanthus emblica* L. by using the UPLC-MS^n^ and network pharmacology methods. 110 compounds were obtained from UPLC-MS^n^ and the characteristic fragmentations were summarized. We found that hydrolysable tannins were the main components of the tannin fraction of *Phyllanthus emblica* L. Then, a network pharmacology method was used to explore the biomarkers for quality control of the tannin fraction of *Phyllanthus emblica* L., gallic acid, punicalagin A, punicalagin B, methyl gallate, geraniin, corilagin, chebulinic acid, chebulagic acid, and ellagic acid were filter as the biomarkers. Animal experiments proved these 9 compounds were proper biomarkers, because we generally believe that the ingredients those are absorbed into the blood are effective. Finally, a simple method for simultaneously measuring the contents of 9 biomarkers was established using HPLC-DAD. This method does not require high equipment, and it is suitable for promotion. The method developed in our study also provides a scientific foundation for the study of anticancer effective substances of the tannin fraction of *Phyllanthus emblica* L.

## Figures and Tables

**Table 1:** The UPLC-MS^n^ data and compound names of the 110 peaks.

Peak no.	*t*_R_ (min)	Molecular formula	[M**−**H]^**−**^	ppm	Negative mode	Identification
1^d^	0.75	C_7_H_8_O_7_	203.0186	−0.18	MS^1^ : 203.0186 [M−H]^−^, MS^2^ : 159.0324 [M−CO_2_−H]^−^	2-oxo-3-carboxyadipic acid
2^d^	0.86	C_6_H_12_O_6_	179.0562	0.25	MS^1^ : 179.0562 [M−H]^−^, MS^2^ : 101.0242 [C_4_H_5_O_3_]^−^, 89.0242 [C_3_H_5_O_3_]^−^, 71.01382 [C_3_H_2_O_2_]^−^	Glucose
3^a^	0.89	C_6_H_10_O_8_	209.0378	0.33	MS^1^ : 209.0378 [M−H]^−^, MS^2^ : 191.0267 [M−H_2_O−H]^−^, 147.0304 [M−H_2_O−CO_2_−H]^−^	Mucic acid
4^a^	0.93	C_6_H_8_O_7_	191.0264	−0.14	MS^1^ : 191.0264 [M−H]^−^, 383.0234 [2M−H]^−^ MS^2^ : 147.0304 [M−CO_2_−H]^−^, 119.0221 [M−CO_2_−CO−H]^−^	Mucic acid lactone
5^a^	0.97	C_4_H_6_O_5_	133.0131	0.23	MS^1^ : 133.0131 [M−H]^−^, MS^2^ : 115.0321 [M−H_2_O−H]^−^	Malic acid
6^e^	1.01	C_13_H_16_O_10_	331.0659	0.34	MS^1^ : 331.0659 [M−H]^−^, 663.1325 [2M−H]^−^, MS^2^ : 169.0145 [galloyl]	6-O-galloylglucose
7^a^	1.05	C_13_H_14_O_12_	361.0406	0.22	MS^1^ : 361.0406 [M−H]^−^, MS^2^ : 209.0378 [M−galloyl−H]^−^, 191.0264 [M–galloyl–H_2_O–H]^−^	Mucic acid 2-O-gallate
8^b^	1.13	C_14_H_12_O_11_	355.0345	−0.55	MS^1^ : 355.0345 [M−H]^−^, MS^2^ : 331.0666	Chebulic acid
9^e^	1.17	C_13_H_16_O_10_	331.0659	0.44	MS^1^ : 331.0659 [M−H]^−^, 663.1325 [2M−H]^−^, MS^2^ : 169.0145 [galloyl]	2-O-galloylglucose
10^b^	1.20	C_20_H_18_O_16_	513.0589	0.33	MS^1^ : 513.0589 [M−H]^−^, MS^2^ : 361.0444 [M−H−galloyl]^−^, 209.0344 [M−H−2 galloyl]^−^	Mucic acid digallate
11^d^	2.57	C_6_H_8_O_7_	191.0198	0.93	MS^1^ : 191.0198 [M−H]^−^, 383.0234 [2M−H]^−^, MS^2^ : 147.1211 [M−CO_2_−H]^−^, 129.0392 [M−CO_2_−H_2_O−H]^−^	Citric acid
12^a^	1.25	C_13_H_12_O_11_	343.0305	−0.29	MS^1^ : 343.0305 [M−H]^−^, MS^2^ : 191.0198 [M−galloyl−H]^−^	Mucic acid lactone gallate
13^e^	1.97	C_13_H_16_O_10_	331.0659	0.27	MS^1^ : 331.0659 [M−H]^−^, 663.1325 [2M−H]^−^, MS^2^ : 169.0145 [galloyl]	1-O-galloylglucose
14^a^	2.16	C_13_H_12_O_11_	343.0305	0.77	MS^1^ : 343.0305 [M−H]^−^, MS^2^ : 191.0198 [M−galloyl−H]^−^	Mucic acid lactone gallate
15^e^	2.32	C_7_H_6_O_5_	169.0122	−0.38	MS^1^ : 169.0122 [M−H]^−^, MS^2^ : 125.0338 [M−CO_2_−H]^−^	Gallic acid
16^a^	2.43	C_14_H_16_O_12_	375.0558	−0.72	MS^1^ : 375.0558 [M−H]^−^, MS^2^ : 223.0434 [M−galloyl−H]^−^	Mucic acid methyl ester gallate
17^b^	2.50	C_20_H_18_O_16_	513.0589	0.66	MS^1^ : 513.0589 [M−H]^−^, MS^2^ : 361.0444 [M−H−galloyl]^−^, 209.0344 [M−H−2 galloyl]^−^	Mucic acid digallate
18^a^	2.57	C_14_H_16_O_12_	375.0558	0.90	MS^1^ : 375.0558 [M−H]^−^, MS^2^ : 345.0323 [M−H−2CH_3_]^−^, 331.0711	Mucic acid methyl ester gallate
19^b^	2.69	C_20_H_18_O_16_	513.0589	−0.33	MS^1^ : 513.0589 [M−H]^−^, MS^2^ : 361.0444 [M−H−galloyl]^−^, 209.0344 [M−H−2 galloyl]^−^	Mucic acid digallate
20^a^	2.89	C_14_H_16_O_12_	375.0558	−0.48	MS^1^ : 375.0558 [M−H]^−^, MS^2^ : 345.0323 [M−H−2CH_3_]^−^, 331.0711	Mucic acid methyl ester gallate
21^b^	3.11	C_20_H_18_O_16_	513.0589	0.78	MS^1^ : 513.0589 [M−H]^−^, MS^2^ : 361.0444 [M−H−galloyl]^−^, 209.0344 [M−H−2galloyl]^−^	Mucic acid digallate
22^a^	3.24	C_14_H_16_O_12_	375.0558	0.92	MS^1^ : 375.0558 [M−H]^−^, MS^2^ : 345.0323[M−H−2CH_3_]^−^, 331.0711	Mucic acid methyl ester gallate
23^b^	4.08	C_20_H_16_O_15_	495.0405	−0.93	MS^1^ : 495.0405 [M−H]^−^, MS^2^ : 343.0356 [M−H−galloyl]^−^, 191.0223 [M−H−2 galloyl]^−^	Mucic acid lactone digallate
24^a^	4.75	C_15_H_18_O_12_	389.0714	0.22	MS^1^ : 389.0714 [M−H]^−^, 779.1529 [2M−H]^−^, MS^2^ : 237.0564 [M−H−galloyl]^−^	Mucic acid dimethyl ester gallate
25^b^	5.10	C_20_H_18_O_16_	513.0589	0.44	MS^1^ : 513.0589 [M−H]^−^, MS^2^ : 361.0444 [M−H−galloyl]^−^, 209.0344 [M−H−2 galloyl]^−^	Mucic acid digallate
26^b^	5.19	C_21_H_20_O_16_	527.0667	0.73	MS^1^ : 527.0667 [M−H]^−^, MS^2^ : 375.0557 [M−H−galloyl]^−^, 223.0448 [M−H−2 galloyl]^−^	Mucic acid methyl ester digallate
27^b^	5.41	C_20_H_20_O_14_	483.0769	−0.92	MS^1^ : 483.0769 [M−H]^−^, 967.1610 [2M−H]^−^ MS^2^ : 331.0712 [M−H−galloyl]^−^, 271.0523, 169.0139	1, 4-di-O-galloylglucose
28^a^	4.79	C_11_H_10_O_9_	285.0241	0.4	MS^1^ : 285.0250 [M−H]^−^, 571.0583 [2M−H]^−^, MS^2^ : 133.0122 [M−H−galloyl]^−^	Malic acid gallate
29^e^	5.89	C_14_H_16_O_10_	343.0659	0.38	MS^1^ : 343.0659 [M−H]^−^, MS^2^ : 255.0522 [M−H−2CO_2_]^−^, 169.0141	3-Galloylquinic acid
30^b^	6.17	C_27_H_26_O_20_	669.0933	−0.65	MS^1^ : 669.0933 [M−H]^−^, MS^2^ : 337.0199 [M−H−galloyl−H_2_O−Hex]^−^	Phyllanemblinin D
31^b^	6.44	C_20_H_20_O_14_	483.0769	0.89	MS^1^ : 483.0769 [M−H]^−^, 967.1610 [2M−H]^−^MS^2^ : 331.0712 [M−H−galloyl]^−^, 271.0523, 169.0139	1, 6-di-O-galloylglucose
32^b^	6.52	C_27_H_26_O_20_	669.0933	−0.19	MS^1^ : 669.0933 [M−H]^−^, MS^2^ : 337.0199 [M−H−galloyl−H_2_O−Hex]^−^	Phyllanemblinin E
33^e^	6.77	C_15_H_18_O_9_	341.0867	−0.28	MS^1^ : 341.0867 [M−H]^−^, MS^2^ : 297.0989 [M−H−CO_2_]^−^, 179.0234 [M−H-glc]^−^	Caffeic acid-3-glucoside
34^b^	6.88	C_14_H_14_O_11_	357.0452	−0.23	MS^1^ : 357.0452 [M−H]^−^, 715.5998 [2M−H]^−^, MS^2^ : 205.0332 [M−H−galloyl]^−^	Mucic acid lactone methyl ester digallate
35^b^	3.84	C_48_H_28_O_30_	1083.0581	0.99	MS^1^ : 1083.0581 [M−H]^−^, MS^2^ : 541.0836 [M−2H]^2−^, 781.3025 [M−H−HHDP]^−^, 300.9921	Punicalagin A
36^b^	5.90	C_48_H_28_O_30_	1083.0581	0.34	MS^1^ : 1083.0581 [M−H]^−^, MS^2^ : 541.0836 [M−2H]^2−^, 781.3025 [M−H−HHDP]^−^, 300.9921	Punicalagin B
37^b^	7.36	C_14_H_14_O_11_	357.0452	0.92	MS^1^ : 357.0452 [M−H]^−^, 715.5998 [2M−H]^−^, MS^2^ : 205.0433 [M−H−galloyl]^−^	Mucic acid lactone methyl ester digallate
38^b^	7.46	C_21_H_20_O_16_	527.0667	0.66	MS^1^ : 527.0667 [M−H]^−^, MS^2^ : 375.0557 [M−H−galloyl]^−^, 223.0448 [M−H−2 galloyl]^−^	Mucic acid methyl ester digallate
39^b^	7.51	C_33_H_28_O_24_	807.0896	0.71	MS^1^ : 807.0896 [M−H]^−^, MS^2^ : 483.0789 [M−H−Ela]^−^, 331.0189 [M−H−Ela−galloyl]^−^, 169.0143	Mallonin
40^c^	7.91	C_15_H_14_O_7_	305.0655	0.59	MS^1^ : 305.0655 [M−H]^−^, MS^2^ : 215.0098	Gallocatechin
41^b^	8.00	C_33_H_28_O_24_	807.0896	0.49	MS^1^ : 807.0896 [M−H]^−^, MS^2^ : 483.0789 [M−H−Ela]^−^, 331.0189 [M−H−Ela−galloyl]^−^, 169.0143	Mallonin
42^b^	8.03	C_20_H_16_O_15_	495.0405	0.88	MS^1^ : 495.0405 [M−H]^−^, MS^2^ : 343.0356 [M−H−galloyl]^−^, 191.0223 [M−H−2 galloyl]^−^	Mucic acid lactone digallate
43^e^	8.34	C_14_H_10_O_9_	321.0251	−0.46	MS^1^ : 321.0251 [M−H]^−^, MS^2^ : 169.1144 [galloyl]^−^	Digallate
44^b^	8.37	C_27_H_26_O_20_	669.0933	0.81	MS^1^ : 669.0933 [M−H]^−^, MS^2^ : 337.0199 [M−H−galloyl−H_2_O−Hex]^−^	Phyllanemblinin F
45^e^	8.60	C_10_H_12_O_7_	243.1600	0.24	MS^1^ : 243.1600 [M−H]^−^, MS^2^ : 169.0144 [galloyl]^−^, 125.0246	1-O-galloyl-glycerol
46^b^	8.78	C_20_H_20_O_14_	483.0769	0.34	MS^1^ : 483.0769 [M−H]^−^, 967.1610 [2M−H]^−^ MS^2^ : 331.0712 [M−H−galloyl]^−^, 271.0523, 169.0139	3, 6-di-O-galloylglucose
47^b^	8.84	C_46_H_36_O_31_	1083.1156	0.66	MS^1^ : 1083.1156 [M−H]^−^, MS^2^ : 541.0836 [M−2H]^2−^, 781.0341 [M−H−HHDP]^−^, 629.0609 [M−H−HHDP−galloyl]^−^, 301.0115	Putranjivain A
48^b^	9.03	C_21_H_20_O_16_	527.0667	0.71	MS^1^ : 527.0667 [M−H]^−^, MS^2^ : 375.0557 [M−H−galloyl]^−^, 223.0448 [M−H−2 galloyl]^−^	Mucic acid methyl ester digallate
49^b^	9.46	C_41_H_28_O_27_	951.0734	0.26	MS^1^ : 951.0734 [M−H]^−^, MS^2^ : 799.0563 [M−H−galloyl]^−^, 497.0219 [M−H−galloyl−HHDP]^−^, 301.0113	Geraniin
50^b^	9.53	C_41_H_30_O_27_	969.0839	0.22	MS^1^ : 969.0839 [M−H]^−^, MS^2^ : 817.0783 [M−H−galloyl]^−^	Phyllanemblinin C
51^b^	9.72	C_41_H_32_O_28_	971.0996	0.30	MS^1^ : 971.0996 [M−H]^−^, MS^2^ : 953.0906 [M−H−H_2_O]^−^, 935.0800 [M−H−2H_2_O]^−^, 467.0361 [M−2H−H_2_O]^2−^, 300.9911	Neochebulagic acid
52^b^	9.39	C_41_H_30_O_27_	953.0890	0.50	MS^1^ : 953.0890 [M−H]^−^, MS^2^ : 476.0412 [M-2H]^2−^, 300.9983	Terchebin
53^e^	10.14	C_8_H_8_O_5_	183.0259	0.61	MS^1^ : 183.0259 [M−H]^−^, MS^2^ : 169.0064 [M−H−CH_3_]^−^	Methyl gallate
54^b^	10.25	C_27_H_24_O_19_	651.0828	0.11	MS^1^ : 651.0828 [M−H]^−^, MS^2^ : 499.0782[M−H−galloyl]^−^	Chebulanin
55^b^	10.33	C_47_H_34_O_32_	1109.0949	0.03	MS^1^ : 1109.0949 [M−H]^−^, MS^2^ : 957.0466 [M−galloyl−H]^−^, 655.0497 [M−galloyl−HHDP−H]^−^, 300.9091	Elaeocarpusin
56^b^	10.48	C_27_H_24_O_18_	635.0878	0.06	MS^1^ : 635.0878 [M−H]^−^, MS^2^ : 465.0679 [M−galloyl−H_2_O−H]^−^, 313.0560 [M−H−galloyl−galloyl–H_2_O]^−^, 169.1045	Trigalloylglucose
57^b^	10.64	C_41_H_30_O_26_	937.0941	0.09	MS^1^ : 937.0941 [M−H]^−^, MS^2^ : 785.0648 [M−galloyl−H]^−^, 633.0566 [M−2 galloyl−H]^−^, 331.0516 [M−H−2 galloyl−HHDP−H]^−^, 300.9416	Punicafolin
58^b^	10.56	C_41_H_28_O_26_	935.0785	0.31	MS^1^ : 935.0785 [M−H]^−^, MS^2^ : 783.0648 [M−galloyl−H]^−^	Casuarinin
59^b^	10.73	C_27_H_22_O_18_	633.0728	0.92	MS^1^ : 633.0728 [M−H]^−^, MS^2^ : 463.0511 [M−galloyl−H]^−^, 301.0221	Corilagin
60^b^	10.73	C_27_H_22_O_18_	633.0738	0.39	MS^1^ : 633.0738 [M−H]^−^, MS^2^ : 481.0511 [M−galloyl−H]^−^, 331.0598 [M−HHDP−H]^−^, 300.9601	Phyllanemblinin B
61^b^	10.73	C_27_H_22_O_18_	633.0722	0.34	MS^1^ : 633.0722 [M−H]^−^, MS^2^ : 481.0511 [M−galloyl−H]^−^, 331.0598 [M−HHDP−H]^−^, 300.9601	Isostrictinin
62^b^	11.14	C_20_H_16_O_15_	495.0405	0.66	MS^1^ : 495.0405 [M−H]^−^, MS^2^ : 343.0356 [M−H−galloyl]^−^, 191.0223 [M−H−2 galloyl]^−^	Mucic acid lactone digallate
63^b^	11.25	C_33_H_26_O_24_	805.0564	0.66	MS^1^ : 805.0564 [M−H]^−^, MS^2^ : 653.0432 [M−galloyl−H]^−^	Mallonin
64^b^	11.65	C_27_H_24_O_18_	635.0878	−0.39	MS^1^ : 635.0878 [M−H]^−^, MS^2^ : 465.0679 [M−H−galloyl−H_2_O]^−^, 313.0560 [M−H−2 galloyl−H_2_O]^−^, 169.1045	Trigalloylglucose
65^b^	11.88	C_27_H_20_O_17_	615.0616	0.09	MS^1^ : 615.0616 [M−H]^−^, MS^2^ : 463.0506 [M−H−galloyl]^−^, 177.0534 [M−H−galloyl−THBDF]^−^	Phyllanemblinin A
66^b^	11.92	C_34_H_26_O_22_	785.0831	0.01	MS^1^ : 785.0831 [M−H]^−^, MS^2^ : 633.0726 [M−H−galloyl]^−^, 463.0685 [M−H−2 galloyl−H_2_O]^−^, 300.9482	Digalloyl-HHDP-glucose
67^c^	12.31	C_15_H_14_O_6_	289.0708	0.22	MS^1^ : 289.0708 [M−H]^−^, MS^2^ : 275.0192 [M−H−CH_2_]^−^, 215.0094	Epicatechin
68^e^	12.35	C_20_H_16_O_13_	463.0507	0.11	MS^1^ : 463.0507 [M−H]^−^, 927.1101 [2M−H]^−^, MS^2^ : 300.9982 [M−H−Hex]^−^	Ellagic acid hexose
69^b^	12.47	C_41_H_30_O_27_	953.0890	0.51	MS^1^ : 953.0890 [M−H]^−^, MS^2^ : 476.0412 [M−2H]^2−^, 300.9983	Chebulinic acid
70^b^	12.47	C_41_H_30_O_27_	953.0890	0.62	MS^1^ : 953.0890 [M−H]^−^, MS^2^ : 476.0412 [M−2H]^2−^, 300.9983	Chebulagic acid
71^b^	12.88	C_34_H_26_O_22_	785.0831	−0.71	MS^1^ : 785.0831 [M−H]^−^, MS^2^ : 633.0726 [M−H−galloyl]^−^, 463.0685 [M−H−2 galloyl−H_2_O]^−^, 300.9922	Digalloyl-HHDP-glucose
72^e^	13.00	C_19_H_14_O_12_	433.0401	0.66	MS^1^ : 433.0401 [M−H]^−^, 867.0895 [2M−H]^−^, MS^2^ : 300.9991 [M−H−pent]^−^	Ellagic acid pentose
73^c^	13.23	C_15_H_14_O_6_	289.0716	−0.11	MS^1^ : 289.0716 [M−H]^−^, MS^2^ : 241.0354 [M−H−2CH_3_−H_2_O]^−^, 215.0094	Catechin
74^b^	13.73	C_34_H_26_O_22_	785.0831	0.19	MS^1^ : 785.0831 [M−H]^−^, MS^2^ : 633.0726 [M−H−galloyl]^−^, 463.0685 [M−H−2 galloyl−H_2_O]^−^, 300.9911	Digalloyl-HHDP-glucose
75^b^	13.80	C_34_H_28_O_22_	787.0988	0.29	MS^1^ : 787.0988 [M−H]^−^, MS^2^ : 483.0785 [M−H−2 galloyl]^−^	1, 2, 3, 6-tetra-O-galloylglucose
76^b^	14.03	C_41_H_30_O_26_	937.0941	−0.13	MS^1^ : 937.0941 [M−H]^−^, MS^2^ : 785.0848 [M−H−galloyl]^−^, 633.0737 [M−H−2 galloyl]^−^, 481.0411[M−H−3 galloyl]^−^, 468.0442 [M−2H]^2−^, 300.9991	Trigalloyl-HHDP-glucose
77^c^	14.06	C_28_H_24_O_16_	615.0980	0.51	MS^1^ : 615.0980 [M−H]^−^, MS^2^ : 463.0893 [M−H−galloyl]^−^	2′-O-galloylhyperin
78^b^	14.12	C_34_H_22_O_22_	781.0518	0.11	MS^1^ : 781.0518 [M−H]^−^, MS^2^ : 629.0521 [M−H−galloyl]^−^, 477.0443 [M−H−2 galloyl]^−^, 175.0551 [M−H−2 galloyl−HHDP]^−^, 300.9559	Emblicanin A
79^b^	10.40	C_48_H_32_O_32_	1119.0792	0.19	MS^1^ : 1119.0792 [M−H]^−^, MS^2^ : 967.0893 [M−H−galloyl]^−^	Mallotusinic acid
80^e^	14.53	C_19_H_14_O_12_	433.0401	0.17	MS^1^ : 433.0401 [M−H]^−^, 867.0895 [2M−H]^−^, MS^2^ : 300.9991 [M−H−pent]^−^	Ellagic acid pentose
81^c^	17.20	C_28_H_24_O_15_	599.1046	−0.22	MS^1^ : 599.1046 [M−H]^−^, MS^2^ : 285.0393 [M−galloylgalactoside−H]^−^, 153.01813	Kaempferol-3-(6″-galloylgalactoside)
82^c^	14.86	C_15_H_12_O_5_	271.0611	0.92	MS^1^ : 271.0611 [M−H]^−^, MS^2^ : 177.0197 [M−C_6_H_7_O−H]^−^, 151.0035 [M−C_6_H_7_O−2CH_3_−H]^−^, 119.0012 [M−C_6_H_7_O−2CH_3_−2CH_4_– H]^−^	Naringenin
83^e^	15.02	C_20_H_16_O_12_	447.0558	0.62	MS^1^ : 447.0558 [M−H]^−^, MS^2^ : 300.9991	Ellagic acid deoxyhexose
84^c^	15.10	C_21_H_20_O_12_	463.0871	−0.15	MS^1^ : 463.0871 [M−H]^−^, MS^2^ : 301.0312 [M−H−hexose] ^−^	Quercetin hexose
85^e^	15.22	C_14_H_6_O_8_	300.9978	−0.71	MS^1^ : 300.9978 [M−H]^−^, MS^2^ : 283.2637 [M−H−H_2_O]^−^, 273.0035 [M−H−CO]^−^, 229.0137 [M−H−CO−CO_2_]^−^	Ellagic acid
86^b^	15.49	C_41_H_26_O_25_	917.0679	0.66	MS^1^ : 917.0679 [ M−H]^−^ MS^2^ : 765.0313 [M−galloyl−H]^−^, 463.0335 [M−H−galloyl−HHDP]^−^, 300.9091	Mallotusinin
87^c^	15.97	C_15_H_14_O5	273.0769	0.92	MS^1^ : 273.0769 [M−H]^−^, MS^2^ : 215.0100	Epiafzelechin
88^b^	16.17	C_41_H_30_O_26_	937.0941	−0.39	MS^1^ : 937.0941 [M−H]^−^, MS^2^ : 785.0848 [M−H−galloyl]^−^, 633.0737 [M−H−2 galloyl]^−^, 481.0411 [M−H−3 galloyl]^−^, 468.0442 [M−2H]^2−^, 300.9922	Trigalloyl-HHDP-glucose
89^e^	16.21	C_9_H_10_O_5_	197.0444	0.09	MS^1^ : 197.0444 [M−H]^−^, MS^2^ : 153.322 [M−CO_2_−H]^−^	Vanillylmandelic acid
90^c^	16.24	C_21_H_20_O_11_	447.0921	0.01	MS^1^ : 447.0921 [M−H]^−^, MS^2^ : 300.9988 [M−H−deoxyhex]^−^	Quercetin deoxyhexose
91^c^	16.24	C_21_H_20_O_11_	447.0927	0.44	MS^1^ : 447.0921[M−H]^−^, MS^2^ : 287.0545 [M−H−Glc]^−^	Luteolin-7-galactoside
92^e^	16.68	C_15_H_8_O_8_	315.0147	−0.72	MS^1^ : 315.0147 [M−H]^−^, MS^2^ : 300.0991 [M−H−CH_3_]^−^, 212.9015 [M−H−CH_3_−2CO_2_]^−^	3-O-Methylellagic acid
93^d^	16.91	C_12_H_20_O_5_	243.1227	0.41	MS^1^ : 243.1227 [M−H]^−^, MS^2^ : 225.1125 [M−H−H_2_O]^−^, 207.5586 [M−H−2H_2_O]^−^, 133.2019 [M−H−2H_2_O−2CH_3_−CO_2_]^−^	Methyl 5, 10-dihydroxy-10-methoxydeca-6, 8-dienoate
94^b^	17.36	C_29_H_36_O_8_	511.2326	0.38	MS^1^ : 511.2326 [M−H]^−^, MS^2^ : 468.2119 [M−CH_3_−CO−H]^−^, 425.1767 [M−2CH_3_−2CO−H]^−^	Mallotojaponin C
95^c^	17.56	C_21_H_20_O_10_	431.0983	-0.15	MS^1^ : 431.0983 [M−H]^−^, MS^2^ : 416.0093 [M−H−CH_3_]^−^	Vitexin
96^e^	18.50	C_16_H_10_O_8_	329.0302	−0.19	MS^1^ : 329.0302 [M−H]^−^, MS^2^ : 255.2328 [M−H−CO_2_−2CH_3_]^−^	3, 4-di-*O*-methylellagic acid
97^d^	18.56	C_10_H_18_O_4_	201.1132	−0.18	MS^1^ : 201.1132 [M−H]^−^, MS^2^ : 183.1023 [M−H−H_2_O]^−^	2-hydroxy-4-oxo-decanoic acid
98^c^	18.80	C_30_H_28_O_13_	595.1446	−0.13	MS^1^ : 595.1446 [M−H]^−^, MS^2^ : 287.0382 [M−H−coumaroylhexose]^−^	Eriodictyol coumaroylhexose
99^c^	19.06	C_30_H_28_O_13_	595.1446	0.19	MS^1^ : 595.1446 [M−H]^−^, MS^2^ : 287.0382 [M−H−coumaroylhexose]^−^	Eriodictyol coumaroylhexose
100^d^	19.20	C_12_H_20_O_4_	227.1288	0.14	MS^1^ : 227.1288 [M−H]^−^, MS^2^ : 183.0382 [M−H−CO_2_]^−^, 157.1079 [M−H−CO_2_−CH = CH]^−^	Traumatic acid
101^b^	20.25	C_34_H_20_O_22_	779.0362	0.01	MS^1^ : 779.0362 [M−H]^−^, MS^2^ : 477.0342 [M−H– HHDP]^−^, 300.9809	Emblicanin B
102^c^	20.56	C_28_H_24_O_14_	583.1082	0.91	MS^1^ : 583.1082 [M**−**H]^−^ MS^2^ : 431.0984 [M**−**H**−**galloyl]^−^, 331.0561	2″-O-galloylisovitexin
103^d^	22.31	C_11_H_20_O_4_	215.1277	0.49	MS^1^ : 215.1277 [M**−**H]^−^, MS^2^ : 197.1181 [M**−**H**−**H_2_O]^−^, 153.1286 [M**−**H**−**H_2_O**−**CO_2_]^−^	5-hydroxy-3-methoxydec-2-enoic acid
104^b^	22.54	C_25_H_30_O_8_	457.1856	0.14	MS^1^ : 457.1856 [M**−**H]^−^, MS^2^ : 414.1181 [M**−**H**−**CH_3_CO]^−^, 371.1286 [M**−**H**−**2CH_3_CO]^−^	Mallotojaponin B
105^d^	25.56	C_18_H_28_O_3_	291.1954	0.66	MS^1^ : 291.1954 [M**−**H]^−^, MS^2^ : 247.0332 [M**−**H**−**CO_2_]^−^	Licanic acid
106^d^	26.07	C_18_H_36_O_4_	315.2535	−0.24	MS^1^ : 315.2535 [M**−**H]^−^, MS^2^ : 297.1526 [M**−**H**−**H_2_O]^−^, 253.1221 [M**−**H**−**H_2_O**−**CO_2_]^−^	Dihydroxystearic acid
107^d^	26.24	C_18_H_30_O_3_	293.2111	−0.29	MS^1^ : 293.2111 [M**−**H]^−^, MS^2^ : 275.2159 [M**−**H**−**H_2_O]^−^, 231.1441 [M**−**H**−**H_2_O**−**CO_2_]^−^	9-hydroxyoctadeca-5, 10, 12-trienoic acid
108^d^	27.01	C_18_H_36_O_2_	283.2641	0.33	MS^1^ : 283.2641 [M**−**H]^−^, MS^2^ : 265.1576 [M**−**H**−**H_2_O]^−^, 237.0132 [M**−**H**−**H_2_O**−**CO]^−^	3-Hydroxyoctadecanal
109^d^	28.73	C_30_H_48_O_3_	455.3519	0.29	MS^1^ : 455.3519 [M**−**H]^−^, MS^2^ : 401.0874 [M**−**H**−**CO_2_]^−^	Ursolic acid
110^d^	30.71	C_16_H_32_O_2_	255.2318	0.33	MS^1^ : 255.2318 [M**−**H]^−^, MS^2^ : 211.0521 [M**−**H**−**CO_2_]^−^	Palmitic acid

a: mucic acid, b: hydrolysable tannin, c: flavonoids, d: fatty acid, and e: phenolic acids.

**Table 2:** Calibration curves of the analytes.

Analytes	Calibration curve	Linear range (ng/mL)	*R* ^*2*^	LOQ (ng/mL)
Gallic acid	*Y* = 2*E* + 06*X* − 30393	4.92∼93.60	0.9991	2.56
Punicalagin A	*Y* = 3*E* + 06*X* − 66826	2.88∼69.70	0.9992	1.44
Punicalagin B	*Y* = 2*E* + 06*X* − 77857	5.27∼125.50	0.9993	1.03
Methyl gallate	*Y* = 3*E* + 06*X* − 55968	2.33∼74.55	0.9991	0.46
Geraniin	*Y* = 741189*X* − 29791	6.00∼192.00	0.9994	1.20
Corilagin	*Y* = 844347*X* − 75313	13.75∼440.00	0.9997	0.28
Chebulinic acid	*Y* = 4*E* + 06*X* − 87496	2.74∼87.65	0.9991	1.35
Chebulagic acid	*Y* = 886311*X* − 15698	2.10∼67.20	0.9993	1.05
Ellagic acid	*Y* = 1*E* + 06*X* − 6569.3	0.45∼144.00	0.9993	0.22

**Table 3:** Contents of 9 compounds (*n* = 6).

Analytes	Contents (%)						
	S1	S2	S3	S4	S5	S6	Mean ± SD
Gallic acid	3.42	3.46	3.39	3.44	3.43	3.38	3.42 ± 0.062
Punicalagin A	0.26	0.23	0.23	0.26	0.28	0.29	0.26 ± 0.023
Punicalagin B	0.43	0.44	0.42	0.43	0.4	0.41	0.42 ± 0.013
Methyl gallate	0.45	0.43	0.45	0.46	0.44	0.45	0.45 ± 0.009
Geraniin	1.15	1.19	1.18	1.15	1.2	1.18	1.15 ± 0.019
Corilagin	2.70	2.72	2.74	2.7	2.71	2.72	2.70 ± 0.014
Chebulinic acid	0.44	0.43	0.45	0.42	0.43	0.44	0.44 ± 0.010
Chebulagic acid	1.14	1.16	1.11	1.18	1.15	1.15	1.14 ± 0.021
Ellagic acid	3.21	3.24	3.22	3.26	3.26	3.24	3.21 ± 0.019

## Data Availability

The data used to support the findings of this study are included within the Supplementary Materials.

## Supplementary Materials

Supplementary Materials Table 1: 128 Blood Absorbed Components.
