# Supplementary material for: Phytochemical Analysis Using UPLC-MSn Combined with Network Pharmacology Approaches to Explore the Biomarkers for the Quality Control of the Anticancer Tannin Fraction of Phyllanthus emblica L. Habitat in Nepal
Source: Evid Based Complement Alternat Med. 2021 Mar 25;2021:6623791. doi: 10.1155/2021/6623791 (PMC8018855; doi:10.1155/2021/6623791)
Supplement: Supplementary Materials — Table 1: 128 Blood Absorbed Components. [file 6623791.f1.docx]

**Table1.** 128 Blood Absorbed Components.

| **Peak** **No.** | ***t*_R_ (min)** | **[M** **−** **H]^−^** | **Negative** **Mode** | **Identification** |
| --- | --- | --- | --- | --- |
| **1^d^** | 0.75 | 203.0186 | **MS^1^:** 203.0186 [M – H] ^−^  **MS^2^:** 159.0324 [M – CO_2_ – H] ^−^ | 1-oxo-1,2,4-butanetricarboxylic acid |
| **2^d^** | 0.86 | 179.0562 | **MS^1^:** 179.0562 [M – H]^−^  **MS^2^:** 101.0242 [C_4_H_5_O_3_]^−^ , 89.0242[C_3_H_5_O_3_]^−^ , 71.0138[C_3_H_2_O_2_]^−^ | glucose |
| **3^a*^** | 3.78 | 241.0314 | **MS^1^:** 241.0314 [M – H]^−^,  **MS^2^:** 169.0131 [M – galloyl – H]^−^， | malic acid gallate derivative |
| **4^a*^** | 4.59 | 257.0310 | **MS^1^:** 257.0310 [M – H]^−^,  **MS^2^:** 169.0131 [M – galloyl – H]^−^， | malic acid gallate derivative |
| **5^a*^** | 9.33 | 299.0231 | **MS^1^:** 299.0231 [M – H]^−^,  **MS^2^:** 133.0131 [M – galloyl – H]^−^， | decarboxymalic acid gallate derivative |
| **6^a*^** | 13.52 | 314.0239 | **MS^1^:** 314.0239 [M – H]^−^,  **MS^2^:** 169.01311 [M – galloyl – H]^−^， | decarboxymalic acid gallate derivative |
| **7^a^** | 1.05 | 361.0406 | **MS^1^:** 361.0406 [M – H]^−^  **MS^2^:** 209.0378 [M – galloyl – H]^−^, 191.0264 [M – H_2_O – H]^−^ | mucic acid 2 - gallate |
| **8^a*^** | 1.32 | 376.0382 | **MS^1^:** 376.0382 [M – H]^−^,  **MS^2^:** 209.0378 [M – galloyl – H]^−^ | mucic acid 2-gallate methylation derivative |
| **9^a*^** | 19.26 | 273.0406 | **MS^1^:** 273.0406 [M – H]^−^  **MS^2^:** 209.0378 [M – galloyl – H]^−^ | decarboxymucic acid 2-gallate derivative |
| **10^b^** | 1.13 | 355.0345 | **MS^1^:** 355.0345 [M – H]^−^,  **MS^2^:** 311.0231 [M – CO_2_ – H] ^−^ | chebulic acid |
| **11^b*^** | 3.11 | 311.0321 | **MS^1^:** 311.0231 [M – H]^−^  **MS^2^:** 169.01311 | decarboxychebulic acid derivative |
| **12^b*^** | 13.92 | 223.0122 | **MS^1^:** 223.0122 [M – H]^−^,  **MS^2^:** 169.01311 | decarboxychebulic acid derivative |
| **13^b*^** | 22.04 | 203.0143 | **MS^1^:** 203.0143 [M – H]^−^,  **MS^2^:** 311.0231 [M - CO_2_ - H] ^−^ | chebulic acid hydrolyze derivative |
| **14^e^** | 1.17 | 331.0659 | **MS^1^:** 331.0659 [M – H]^−^, 663.1325 [2M – H]^−^  **MS^2^:** 169.01454 [M – H – glucose] ^−^ | 2-O-galloylglucose |
| **15^a^** | 1.20 | 513.0589 | **MS^1^:** 513.0589 [M – H]^−^  **MS^2^:** 361.0444 [M – H – galloyl] ^−^,  209.0344 [M – H – 152galloyl – 152galloyl] ^−^ | mucic acid digallate |
| **16^d*^** | 4.60 | 152.0198 | **MS^1^:** 152.0198 [M – H]^−^ | decarboxycitric acid derivative |
| **17^a^** | 1.25 | 343.0305 | **MS^1^:** 343.0305 [M – H]^−^  **MS^2^:** 191.0198 [M – galloyl – H]^−^ | mucic acid lactone gallate |
| **18^e^** | 1.97 | 331.0659 | **MS^1^:** 331.0659 [M – H]^−^, 663.1325 [2M – H]^−^  **MS^2^:** 169.01454 [M – H – glucose] ^−^ | 1-O-galloylglucose |
| **19^d*^** | 2.26 | 152.0201 | **MS^1^:** 152.0201 [M – H]^−^, | decarboxyquinic acid derivative |
| **20^a*^** | 2.16 | 299.0305 | **MS^1^:** 299.0305 [M – H] ^−^,  **MS^2^:** 191.0198 [M – galloyl – H]^−^ | decarboxymucic acid lactone gallate derivative |
| **21^e*^** | 2.26 | 108.0132 | **MS^1:^** 108.0132 [M – H] ^−^,  **MS^2^:** 81.0142 [M – COOH] ^−^ | pyrogallol derivative |
| **22^e^** | 2.32 | 169.0122 | **MS^1^:** 169.0122 [M – H]^−^  **MS^2^:** 125.0338 [M – COOH] ^−^ | gallic acid |
| **23^b^** | 3.84 | 1083.0581 | **MS^1^:** 1083.0581 [M – H]^−^,  **MS^2^:** 541.0836 [M – 2H ]^2−^ 389.0726 [M – H – 152 galloyl] ^−^ | punicalagin A |
| **24^b^** | 5.90 | 1083.0581 | **MS^1^:** 1083.0581 [M – H]^−^  **MS^2^:** 541.0836 [M – 2H ]^2−^ 389.0726 [M – H – 152 galloyl] ^−^ | punicalagin B |
| **25^e*^** | 1.38 | 360.0610 | **MS^1^:** 360.0610 [M – H]^−^  **MS^2^:** 183.0566 [M – 176 glucuronic – H]^−^ | methyl gallic acid glucuronide derivative |
| **26^e*^** | 2.40 | 345.0561 | **MS^1^:** 345.0561 [M – H]^−^  **MS^2^:** 169.0806 [M – 176 glucuronic – H]^−^,  125.0433 [M – 176 glucuronic – 44 CO_2_ – H]^−^ | gallic acid glucuronide derivative |
| **27^a^** | 2.50 | 513.0589 | **MS^1^:** 513.0589 [M – H]^−^  **MS^2^:** 361.0444 [M – H – 152 galloyl] ^−^,  209.0344 [M – H – 152 galloyl – 152 galloyl] ^−^ | mucic acid digallate |
| **28^a^** | 2.57 | 375.0558 | **MS^1^:** 375.0558 [M – H]^−^  **MS^2^:**223.0323[M – H –152 galloyl] ^−^ | mucic acid methyl ester gallate |
| **29^a*^** | 7.50 | 285.0342 | **MS^1^:** 285.0342 [M – H]^−^  **MS^2^:** 135.0312 [M – H –152 galloyl] ^−^ | mucic acid methyl ester gallate derivative |
| **30^a^** | 2.69 | 513.0589 | **MS^1^:** 513.0589 [M – H]^−^  **MS^2^:** 361.0444 [M – H – 152 galloyl] ^−^,  209.0344 [M – H –– 152 galloyl – 152 galloyl] ^−^ | mucic acid digallate |
| **31^a^** | 3.11 | 513.0589 | **MS^1^:** 513.0589 [M – H]^−^  **MS^2^:** 361.0444 [M – H –152 galloyl] ^−^,  209.0344 [M – H – 152 galloyl – 152 galloyl] ^−^ | mucic acid digallate |
| **32^a*^** | 5.75 | 345.0714 | **MS^1^:** 345.0714 [M – H]^−^  **MS^2^:** 193.0564 [M – H –152 galloyl] ^−^ | decarboxymucic acid dimethyl ester gallate derivative |
| **33^a^** | 5.10 | 513.0589 | **MS^1^:** 513.0589 [M – H]^−^  **MS^2^:** 361.0444[M – H – 152 galloyl] ^−^,  209.0344 [M – H – 152 galloyl – 152 galloyl] ^−^ | mucic acid digallate |
| **34^a^** | 5.19 | 527.0667 | **MS^1^:** 527.0667 [M – H]^−^  **MS^2^:** 375.0557 [M – H – 152 galloyl] ^−^,  223.0448 [M – H – 152 galloyl – 152 galloyl] ^−^ | mucic acid methyl ester digallate |
| **35^b^** | 5.41 | 483.0769 | **MS^1^:** 483.0769 [M – H]^−^, 967.1610 [2M – H]^−^  **MS^2^:** 331.0712 [M – H – galloyl] ^−^, 271.0523, 169.0139 | 1,4-di-O-galloylglucose |
| **36^a^** | 4.79 | 285.0241 | **MS^1^:** 285.0250 [M – H]^−^, 571.0583 [2M – H]^−^  **MS^2^:** 133.0122 [M – H – 152 galloyl] ^−^ | malic acid gallate |
| **37^a*^** | 3.28 | 463.0122 | **MS^1^:** 463.0122 [M – H]^−^  **MS^2^:** 133.0122 [M – H – 152 galloyl] ^−^ | malic acid gallate glucuronide derivative |
| **38^e*^** | 2.89 | 169.0122 | **MS^1^:** 169.0122 [M – H]^−^ | 3-galloylquinic acid hydrolyzed derivative |
| **39^b^** | 6.17 | 669.0933 | **MS^1^:** 669.0933 [M – H]^−^  **MS^2^:** 337.0199 [M – H – 152galloyl – 18H_2_O – 162Hex] ^−^ | phyllanemblinin D |
| **40^b^** | 6.44 | 483.0769 | **MS^1^:** 483.0769 [M – H]^−^, 967.1610 [2M – H]^−^  **MS^2^:** 331.0712 [M – H – galloyl]-, 271.0523, 169.0139 | 1,6-di-O-galloylglucose |
| **41^b^** | 6.52 | 669.0933 | **MS^1^:** 669.0933 [M – H]^−^  **MS^2^:** 337.0199 [M – H – galloyl – H_2_O – Hex]^−^ | phyllanemblinin E |
| **42^a^** | 6.88 | 357.0452 | **MS^1^:** 357.0452 [M – H]^−^, 715.5998 [2M – H]^−^  **MS^2^:** 205.0332 [M – H – galloyl] ^−^ | mucic acid lactone methyl ester digallate |
| **43^a*^** | 3.16 | 439.0233 | **MS^1^:** 439.0233 [M – H]^−^  **MS^2^:** 357.0452 [M – H – 82 SO_3_H] ^−^ **,** 315.0722 [M – H–82 SO_3_H – CO_2_]^−^ | mucic acid lactone methyl ester digallate [sulfation](D:/Program%20Files%20(x86)/Youdao/Dict/7.5.0.0/resultui/dict/?keyword=hydrogenide) derivative |
| **44^a^** | 7.36 | 357.0452 | **MS^1^:** 357.0452 [M – H]^−^, 715.5998 [2M – H]^−^  **MS^2^:** 205.0433 [M – H – 152 galloyl] ^−^ | mucic acid lactone methyl ester digallate |
| **45^a*^** | 4.61 | 537.0433 | **MS^1^:** 537.0433 [M – H]^−^  **MS^2^:** 357.0452 [M – H – 178glu] ^−^ **,** 315.0722 [M – H– 178glu – CO_2_]^−^ | \| mucic acid lactone methyl ester glucuronide digallate [hydrogenide](D:/Program%20Files%20(x86)/Youdao/Dict/7.5.0.0/resultui/dict/?keyword=hydrogenide)derivative \| \| --- \| |
| **46^a^** | 7.46 | 527.0667 | **MS^1^:** 527.0667 [M – H]^−^  **MS^2^:** 375.0557 [M – H – 152 galloyl]^−^,  223.0448 [M – H – 152 galloyl – 152 galloyl] ^−^ | mucic acid methyl ester digallate |
| **47^b^** | 7.59 | 807.0896 | **MS^1^:** 807.0896 [M – H]^−^  **MS^2^:** 655.0789 [M – H – 152 galloyl]^−^ | mallonin |
| **48^c^** | 7.91 | 305.0655 | **MS^1^:** 305.0655 [M – H]^−^  **MS^2^:** 215.0098[M – H – CH_3_COOH – 2CH_3_]^−^ | gallocatechin |
| **49^a*^** | 8.83 | 407.0405 | **MS^1^:** 407.0405 [M – H]^−^  **MS^2^:** 255.0356 [M – H – 152 galloyl]^−^,103.0332 | decarboxymucic acid lactone digallate derivative |
| **50^e^** | 8.34 | 321.0251 | **MS^1^:** 321.0251 [M – H]^−^  **MS^2^:** 169.1144 [galloyl]^−^ | digallate |
| **51^b^** | 8.37 | 669.0933 | **MS^1^:** 669.0933 [M – H]^−^  **MS^2^:** 337.0199 [M – H – 152galloyl – 18H_2_O – 162Hex] ^−^ | phyllanemblinin F |
| **52^e*^** | 5.53 | 325.1333 | **MS^1^:** 325.1333 [M – H]^−^  **MS^2^:** 243.0144 [M – H – 82SO_3_H] ^−^**,**  125.0246 [M – H – SO_3_H – 2CO_2_ – 2CH_3_] ^−^ | 1-O-galloyl-glycerol sulfation derivative |
| **53^b^** | 8.78 | 483.0769 | **MS^1^:** 483.0769 [M – H]^−^, 967.1610 [2M – H]^−^  **MS^2^:** 331.0712 [M – H – galloyl]^−^, 271.0523, 169.0139 | 3,6-di-O-galloylglucose |
| **54^b^** | 8.84 | 1083.1156 | **MS^1^:** 1083.1156 [M – H]^−^  **MS^2^:** 541.0836 [M – 2H ]^2−^ 389.0726 [M – H – galloyl] ^−^ | putranjivain A |
| **55^a^** | 9.03 | 527.0667 | **MS^1^:** 527.0667 [M – H]^−^  **MS^2^:** 375.0557 [M – H – 152 galloyl] ^−^,  223.0448 [M – H – 152 galloyl – 152 galloyl] ^−^ | mucic acid methyl ester digallate |
| **56^b^** | 9.46 | 951.0734 | **MS^1^:** 951.0734 [M – H]^−^  **MS^2^:** 799.0563 [M – H – 152 galloyl] ^−^,  495.03443 [M – H – 152 galloyl – 286 HHDP] ^−^ | geraniin |
| **57^b*^** | 9.88 | 801.0739 | **MS^1^:** 801.0739 [M – H]^−^  **MS^2^:** 515.0633 [M – H – 286 HHDP] ^−^ | phyllanemblinin C hydrolyzed derivative |
| **58^b^** | 9.72 | 971.0996 | **MS^1^:** 971.0996 [M – H]^−^  **MS^2^:** 953.0906 [M – H – H_2_O]-, 935.0800 [M – H – 2H_2_O] ^−^,  467.0361 [M – 2H – 2H_2_O]^2-^，300.991 | neochebulagic acid derivative |
| **59^b^** | 9.79 | 953.0890 | **MS^1^:** 953.0890 [M – H] ^−^  **MS^2^:** 476.0412 [M-2H]^2−^，300.9983 | terchebin |
| **60^e*^** | 2.33 | 168.0064 | **MS^1^:** 168.0064 [M – H – CH_3_] ^−^ | methyl gallate hydrolyzed derivative |
| **61^e*^** | 8.54 | 361.0222 | **MS^1^:** 361.0222 [M – H] ^−^  **MS^2^:** 183.0259 [M – glu – H] ^−^, 168.0064 [M – H – glu – CH_3_] ^−^ | methyl gallate glucuronide derivative |
| **62^b^** | 10.25 | 651.0828 | **MS^1^:** 651.0828 [M – H]^−^  **MS^2^:** 499.0782[M – H – 152galloyl] ^−^ | chebulanin |
| **63^b^** | 10.33 | 1109.0949 | **MS^1^:** 1109.0949 [M – H]^−^  **MS^2^:** 957.0466 [M – 152 galloyl – H]^−^,  653.0566 [M – 152 galloyl –304 HHDP– H]^−^ | elaeocarpusin |
| **64^b^** | 10.48 | 635.0878 | **MS^1^:** 635.0878 [M – H]^−^  **MS^2^:** 465.0679 [M -152 galloyl-18H_2_O -H] ^–^  313.0560 [M -H – 152 galloyl – 152 galloyl -H_2_O] ^−^，169.1045 | trigalloylglucose |
| **65^b*^** | 13.81 | 785.0648 | **MS^1^:** 785.0648 [M – H]^−^  **MS^2^:** 633.0566 [M – 152 galloyl– H]^−^,  329.0782 [M – H – 152 galloyl –302 HHDP– H] ^−^ | punicafolin hydrolyzed derivative |
| **66^b^** | 10.56 | 935.0785 | **MS^1^:** 937.0941 [M – H]^−^  **MS^2^:** 785.0648 [M – 152galloyl – H]^−^ | casuarinin |
| **67^b^** | 12.47 | 953.0890 | **MS^1^:** 953.0890 [M – H]^−^, **MS^2^:** 476.0412 [M – 2H]^2−^，300.9983 | chebulinic acid |
| **68^b^** | 10.73 | 633.0738 | **MS^1^:** 633.0738 [M – H]^−^  **MS^2^:** 481.0511 [M – 152 galloyl – H]^−^ ,  377.0121 [M – 152 galloyl – 304 HHDP– H]^−^ | phyllanemblinin B |
| **69^b^** | 10.73 | 633.0722 | **MS^1^:** 633.0722 [M – H]^−^  **MS^2^:** 481.0511 [M –152 galloyl – H]^−^ ,  377.0121 [M – 152 galloyl –304 HHDP– H]^−^ | isostrictinin |
| **70^b*^** | 8.44 | 887.0298 | **MS^1^:** 887.0298 [M – H]^−^  **MS^2^:** 805.0564 [M – SO_3_H - H]^−^, 653.0432 [M – SO_3_H - 152 galloyl – H]^−^ | mallonin sulfation derivative |
| **71^b^** | 11.65 | 635.0878 | **MS^1^:** 635.0878 [M – H]^−^  **MS^2^:** 465.0679 [M – H – 152galloyl – 18H_2_O] ^−^,  313.0560 [M – H – 152galloyl – 152galloyl – 18H_2_O] ^−^，169.1045 | trigalloylglucose |
| **72^b*^** | 12.93 | 463.0506 | **MS^1^:** 463.0506 [M – H]^−^  **MS^2^:** 177.0534 [M – H –286THBDF] ^−^ | phyllanemblinin A hydrolyzed derivative |
| **73^b^** | 11.92 | 785.0831 | **MS^1^:** 785.0831 [M – H] ^−^  **MS^2^:** 633.0726 [M – H – 152galloyl] ^−^,  465.0685 [M – H – 152galloyl – 152 galloyl– 18H_2_O] ^−^ | digalloyl-HHDP-glucose |
| **74^c*^** | 9.77 | 467.0433 | **MS^1^:** 467.0433 [M – H]^−^  **MS^2^:** 289.0708 [M – 178glc - H]^−^, 275.0192 [M – H – 14CH_2_]^−^,  215.0094 [M– 60CH_3_COOH – 14CH_2_ – H]^−^ | epicatechin glucuronide derivative |
| **75^e^** | 12.35 | 463.0507 | **MS^1^:** 463.0507 [M – H]^−^, 927.1101 [2M – H]^−^  **MS^2^:** 300.9982 [M – H – 162Hex] ^−^ | ellagic acid hexose |
| **76^b^** | 10.73 | 633.0728 | **MS^1^:** 633.0728 [M – H]^−^  **MS^2^:** 463.0511 [M – galloyl – H]^−^, 301 [M – H-galloyl – H_2_O – Hex]^−^ | corilagin |
| **77^b^** | 12.47 | 953.0890 | **MS^1^:** 953.0890 [M – H]^−^  **MS^2^:** 476.0412 [M – 2H]^2−^，300.9983 | chebulagic acid |
| **78^b^** | 12.88 | 785.0831 | **MS^1^:** 785.0831 [M – H]^−^  **MS^2^:** 633.0726 [M – H – 152galloyl] ^−^,  465.0685 [M – H – 152galloyl – 152galloyl – 18H_2_O] ^−^ | digalloyl-HHDP-glucose |
| **79^e^** | 13.00 | 433.0401 | **MS^1^:** 433.0401 [M – H]^−^ 867.0895 [2M – H]^−^  **MS^2^:** 300.9991 [M – H – 133pent] – | ellagic acid pentose |
| **80^c^** | 13.08 | 477.1027 | **MS^1^:** 477.1027 [M – H]^−^  **MS^2^:** 433.0413 [M –44 CO_2_ – H] ^−^ | isorhamnetin-7-O-glucopyranoside |
| **81^c*^** | 9.77 | 467.0433 | **MS^1^:** 467.0433 [M – H]^−^  **MS^2^:** 289.0708 [M – 178glc - H]^−^, 275.0192 [M – H – 14CH_2_]^−^,  215.0094 [M– 60CH_3_COOH – 14CH_2_ – H]^−^ | catechin glucuronide derivative |
| **87^c*^** | 7.33 | 371.0321 | **MS^1^:** 371.0321 [M – H]^−^  **MS^2^:** 289.0708 [M – 82 SO_3_H – H]^−^, 275.0192 [M – H – 14CH_2_]^−^,  215.0094 [M– 60CH_3_COOH – 14CH_2_ – H]^−^ | catechin sulfation derivative |
| **82^b^** | 13.73 | 785.0831 | **MS^1^:** 785.0831 [M – H]^−^  **MS^2^:** 633.0726 [M – H – 152galloyl] ^−^,  465.0685 [M – H – 152galloyl – 152galloyl – 18H_2_O] ^−^ | digalloyl-HHDP-glucose |
| **83^b*^** | 12.48 | 635.0882 | **MS^1^:** 635.0882 [M – H]^−^  **MS^2^:** 483.0785 [M – H – galloyl] ^−^，  331.0778[M – H – 152galloyl – 152galloyl] ^−^,169.0112 | 1,2,3,6-tetra-O-galloylglucose hydrolyzed derivative |
| **84^b*^** | 15.89 | 669.0893 | **MS^1^:** 669.0893 [M – H]^−^  **MS^2^:** 517.0788 [M – H – 152 galloyl] ^−^ | trigalloyl-glucose hydrolyzed derivative |
| **85^e*^** | 2.56 | 793.0343 | **MS^1^:** 793.0343 [M – H]^−^  **MS^2^:** 615.0980 [M – 178glc – H]^−^,  463.0893 [M – H – 178glc – 152 galloyl] ^−^ | 2’-O-Galloylhyperin hydrolyzed derivative |
| **86^b*^** | 13.66 | 631.0334 | **MS^1^:** 631.0334 [M – H]^−^  **MS^2^:** 479.0893 [M – H – 152 galloyl] ^−^ | 2’-O-Galloylhyperin hydroxylation derivative |
| **87^b*^** | 14.88 | 629.0521 | **MS^1^:** 629.0521 [M – H]^−^  **MS^2^:** 477.0443 [M – H – 152 galloyl] ^−^,  173.0551 [M – H – 152 galloyl – 302 HHDP] ^−^ | emblicanin A hydrolyzed derivative |
| **88^b*^** | 9.53 | 967.0893 | **MS^1^:** 967.0893 [M – H]^−^  **MS^2^:** 665.0988 [M – H – 302 HHDP] ^−^ | mallotusinic acid hydrolyzed derivative |
| **89^e^** | 14.53 | 433.0401 | **MS^1^:** 433.0401 [M – H]^−^ , 867.0895 [2M – H]^−^  **MS^2^:** 300.9991 [M – H – pent] ^−^ | ellagic acid pentose |
| **90^c*^** | 14.79 | 777.1029 | **MS^1^:** 777.1029 [M – H]^−^  **MS^2^:** 599.1046[M – 178glc – H]^−^,  285.0393 [M –6”-galloylgalactoside – H]^−^ , 153.01813 [C_7_H_3_O_4_] ^−^ | kaempferol-3-(6”-galloylgalactoside) glucuronide derivative |
| **91^e^** | 15.02 | 447.0558 | **MS^1^:** 433.0401 [M – H]^−^ 867.0895 [2M – H]^−^  **MS^2^:** 300.9991 | ellagic acid deoxyhexose |
| **92^e^** | 15.22 | 300.9978 | **MS^1^:** 300.9978 [M – H]^−^  **MS^2^:** 283.2637 [M -H-H_2_O]^−^, 273.0035 [M -H-CO]^−^,  229.0137 [M -H-CO-CO_2_]^−^, 210.0485 [M -H-2CO-CO_2_]^−^ | ellagic acid |
| **93^b*^** | 19.98 | 615.0345 | **MS^1^:** 615.0345 M – H]^−^  **MS^2^:** 463.0335 [M – H – 152 galloyl]^−^ | mallotusinin hydrolyzed derivative |
| **94^c*^** | 11.33 | 451.0776 | **MS^1^:** 451.0776 [M – H]^−^  **MS^2^:** 273.0769 [M – 178glc – H]^−^,  215.0100 [M – 178glc –CO_2_ – CH_2_ – H]^−^ | epiafzelechin glucuronide derivative |
| **95^b*^** | 13.81 | 482.0411 | **MS^1^:** 482.0411 [M – H]^−^  **MS^2^:** 300.9991 | trigalloyl-HHDP- hydrolyzed glucose |
| **96^e*^** | 17.34 | 153.3220 | **MS^1^:** 153.3220 [M – H]^−^ | decarboxyvanillylmandelic acid derivative |
| **97^e*^** | 12.78 | 375.0322 | **MS^1^:** 375.0322 [M – H]^−^  **MS^2^:** 197.0444 [M – 178glc – H]^−^, 153.322 [M –CO_2_ – H]^−^ | vanillylmandelic acid glucuronide derivative |
| **98^e*^** | 11.13 | 279.0223 | **MS^1^:** 279.0223 [M – H]^−^  **MS^2^:** 197.0444[M –82 SO_3_H – H]^−^, 153.322 [M –CO_2_ – H]^−^ | vanillylmandelic acid sulfation derivative |
| **99^d*^** | 12.31 | 493.0122 | **MS^1^:** 493.0122 [M – H]^−^  **MS^2^:** 315.0147[M – 178glc – H]^−^, 300.0991 [M – H– 178glc –CH_3_]^−^,  212.9015 [M – H– 178glc – CH_3_ – 2CO_2_]^−^ | 2,3,8-trihydroxy-7-methoxychromeno  [5,4,3-cde]chromene-5,10-dione glucuronide derivative |
| **100^d*^** | 18.12 | 287.0388 | **MS^1^:** 287.0388 [M – H]^−^  **MS^2^:** 259.0176 [M – H –CO]^−^ | Decarboxy-2,3,8-trihydroxy-7-methoxychromeno  [5,4,3-cde]chromene-5,10-dione derivative |
| **101^d*^** | 10.02 | 397.0231 | **MS^1^:** 397.0231 [M – H]^−^  **MS^2^:** 315.0147[M –82 SO_3_H – H]^−^, 300.0991 [M – H–82 SO_3_H –CH_3_]^−^,  212.9015 [M – H–82 SO_3_H – CH_3_ – 2CO_2_]^−^ | 2,3,8-trihydroxy-7-methoxychromeno  [5,4,3-cde]chromene-5,10-dione sulfation derivative |
| **102^d*^** | 18.92 | 259.0176 | **MS^1^:** 259.0176 [M – H]^−^ | decarboxy-2,3,8-trihydroxy-7-methoxychromeno  [5,4,3-cde]chromene-5,10-dione derivative |
| **103^d*^** | 11.27 | 325.1099 | **MS^1^:** 325.1099 [M – H]^−^  **MS^2^:** 243.1227[M –82 SO_3_H – H]^−^, 225.1125 [M – H –82 SO_3_H–H_2_O]^−^,  207.5586 [M – H –82 SO_3_H– 2H_2_O]^−^ | oxododecanedioic acid sulfation derivative |
| **104^d*^** | 17.21 | 215.1022 | **MS^1^:** 215.1022 [M – H]^−^ | decarboxyoxododecanedioic acid derivative |
| **105^d*^** | 20.76 | 187.1227 | **MS^1^:** 186.1227 [M – H]^−^ | decarboxyoxododecanedioic acid derivative |
| **106^b*^** | 17.36 | 467.2213 | **MS^1^:** 467.2213 [M – H]^−^  **MS^2^:** 423.2119 [M– 44 CO_2_ – H]^−^ , 408.1767 [M –15 CH_3_ – 44 CO_2_ – H]^−^ | decarboxymallotojaponin C derivative |
| **107^e*^** | 14.19 | 507.0112 | **MS^1^:** 507.0112 [M – H]^−^  **MS^2^:** 329.0302 [M – 178glc – H]^−^,  255.2328 [M – H– 178glc – CO_2_ – 2CH_3_] ^−^ | 2,3-di-*O*-methylellagic acid glucuronide derivative |
| **108^e*^** | 12.12 | 411.0214 | **MS^1^:** 411.0214 [M – H]^−^  **MS^2^:** 329.0302 [M –82 SO_3_H – H]^−^,  255.2328 [M – H –82 SO_3_H – CO_2_ – 2CH_3_] ^−^ | 2,3-di-*O*-methylellagic acid sulfation derivative |
| **109^d*^** | 23.01 | 183.0122 | **MS^1^:** 183.0122 [M – H]^−^  **MS^2^:** 155.1023 [M – H –CO]^−^ | sebacic acid dehydration derivative |
| **110^d*^** | 27.23 | 287.0382 | **MS^1^:** 287.0382 [M – H]^−^ | eriodictyol coumaroylhexose hydrolyzed derivative |
| **111^d^** | 19.20 | 227.1288 | **MS^1^:** 227.1288 [M – H]^−^  **MS^2^:** 183.0382 [M -H–CO_2_] ^−^, 165.1238 [M – H – CO_2_ – H_2_O] ^−^ | traumatic acid |
| **112^d*^** | 22.09 | 219.2302 | **MS^1^:** 219.2302 [M – H]^−^ | decarboxyabscisic acid derivative |
| **113^d*^** | 26.32 | 191.2302 | **MS^1^:** 263.0346 [M – H]^−^  **MS^2^:** 219.0276 [M -H–CO_2_] ^−^, 204.0304 [M – H – CH_3_] ^−^ | decarboxyabscisic acid derivative |
| **114^b^** | 20.25 | 779.0362 | **MS^1^:** 779.0362 [M – H]^−^  **MS^2^:** 475.0342 [M – H– HHDP] ^−^ | emblicanin B |
| **115^d^** | 20.42 | 215.1277 | **MS^1^:** 215.1277 [M – H]^−^  **MS^2^:** 197.1181 [M – H –H_2_O]^−^ , 153.1286 [M – H –H_2_O –CO_2_]^−^ | undecanedioic acid |
| **116^e^** | 20.52 | 313.0930 | **MS^1^:** 313.0930 [M – H]^−^  **MS^2^:** 161.0423 [C_6_H_9_O_5_]^−^ | glucovanillin |
| **117^e^** | 20.56 | 583.1082 | **MS^1^:** 583.1082 [M – H]^−^  **MS^2^:** 431.0984 [M – H – 152 galloyl] ^−^,  331.0561 [M – H – 152 galloyl – C_3_H_6_O_3_ – 2CH_3_] ^–^ | 2”-O-Galloylisovitexin |
| **118^d^** | 22.31 | 215.1277 | **MS^1^:** 215.1277 [M – H]^−^  **MS^2^:** 197.1181 [M – H – H_2_O]^−^ ,153.1286 [M – H –H_2_O – CO_2_]^−^ | undecanedioic acid |
| **119^b^** | 22.54 | 457.1856 | **MS^1^:** 457.1856 [M – H]^−^  **MS^2^:** 414.1181 [M – H – CH_3_CO]^−^ ,371.1286 [M – H – CH_3_CO – CH_3_CO]^−^ | mallotojaponin B |
| **120^d*^** | 26.99 | 247.2098 | **MS^1^:** 247.2098 [M – H]^−^  **MS^2^:** 219.0453 [M – H –H_2_O – CO]^−^ | decarboxylicanic acid derivative |
| **121^d^** | 26.07 | 315.2535 | **MS^1^:** 315.2535 [M – H]^−^  **MS^2^:** 297.1526 [M – H –H_2_O]^−^,253.1221 [M – H –H_2_O – CO_2_]^−^ | dihydroxystearic acid |
| **122^d*^** | 30.33 | 249.2098 | **MS^1^:** 249.2098 [M – H]^−^,  **MS^2^:** 231.1441 [M – H –H_2_O]^−^ | decarboxykamlolenic acid derivative |
| **123^d*^** | 30.66 | 425.3312 | **MS^1^:** 425.3312.3312 [M – H]^−^  **MS^2^:** 381.2527 [M – H – CO_2_]^−^ | decarboxynigranoic acid derivative |
| **124^d*^** | 28.67 | 239.2245 | **MS^1^:** 239.2245 [M – H]^−^  **MS^2^:** 211.0097 [M – H – CH_2_= CH_2_]^−^ | decarboxystearic acid derivative |
| **125^f^** | 29.19 | 455.3519 | **MS^1^:** 455.3519 [M – H]^−^  **MS^2^:** 401.0874 [M – H –CO_2_]^−^ | oleanic acid |
| **126^d^** | 30.71 | 255.2318 | **MS^1^:** 255.2318 [M – H]^−^  **MS^2^:** 237.2215 [M – H –H_2_O]^−^ | palmitic acid |
| **127^d^** | 31.13 | 281.2475 | **MS^1^:** 281.2475 [M – H]^−^, 141.0170 [2M – H]^−^  **MS^2^:** 255.2324 [M – H – 2CH_3_]^−^, 236.9104 [M – H – CO_2_]^−^ | oleic acid |
| **128^d^** | 31.90 | 311.2950 | **MS^1^:** 311.2950 [M – H]^−^  **MS^2^:** 237.2215 [M – H –H_2_O]^−^ | eicosanoic acid |

a: mucic acids, b: hydrolysable tannins, c: flavonoids, e: phenolic acids, d: fatty acid ,f: other components，* metabolite

In view of the blood-absorbed components taking effects, the potential quality control markers screened by network pharmacology were further analyzed and confirmed in rat plasma after oral administration of the tannin fraction of *Phyllanthus emblica* L. by UPLC-MS^n^.
